# Prospective evaluation of dose rate effects on acute radiation toxicities and quality of life in head and neck cancer

**DOI:** 10.1186/s43046-026-00375-6

**Published:** 2026-07-09

**Authors:** Ahmed Adel Ahmed, Wahib M Attia, O ELemary, Mohamed Abouegylah

**Affiliations:** 1 Suez Canal University, Ismailia, Egypt; 2 Clinical Oncology Department, Faculty of Medicine, Alexandria University, Alexandria, Egypt

**Keywords:** Dose rate, Head and neck cancer, IMRT, Acute toxicity, QLQ-H&N 35

## Abstract

**Purpose:**

This study evaluates the influence of different dose rates on the radiation-related adverse events.

**Materials and methods:**

The study involved 60 patients divided into three groups, each receiving different dose rates of the Flattening Filter-Free (FFF) 6MV IMRT technique: 20 patients at 600, 20 at 1000, and 20 at 1400. Acute adverse events were monitored weekly. The EORTC-translated QLQ-H&N 35 questionnaire was used to assess health-related quality of life (QoL) according to established EORTC criteria during radiotherapy.

**Results:**

During the initial phase of radiotherapy treatment, no significant differences were noted in the prevalence of conditions such as mucositis and weight loss among the treatment groups. However, weight loss showed a significant variation (*p* = 0.028) at third week for the 600-dose rate group. From the fourth to fifth week, no notable differences emerged for mucositis and xerostomia among groups, while increase radiation dose rate was correlated with significant rise in dermatitis and dysphagia (*p* = 0.020 and *p* = 0.036, respectively). Similarly, from week five to six and from week six to seven, no significant differences were observed for mucositis. Yet significant increase in xerostomia and other conditions was evident with rising radiation dose rate. The evaluation of the QLQ-H&N 35 for head and neck cancer patients revealed a numerical scale for symptom severity, from 1 (not at all) to 4 (very much). While differences in symptom intensity across dose rates were noted, they did not reach statistical significance.

**Conclusion:**

The dose rate appears to influence patient health outcomes, although it does not seem to affect the QoL during the period of radiotherapy course treatment.

## Introduction

Head and neck cancer (HNC) represents a heterogeneous group of neoplasms that originate from the oral cavity, larynx, nasopharynx, oropharynx, or hypopharynx [1]. Therapeutic interventions typically involve a multi-faceted strategy integrating various modalities including surgical procedures, radiotherapeutic techniques, and chemotherapy regimens [2]. Consequently, due to treatment side effects, patients with head and neck cancer (HNC) often require multidisciplinary management during and after treatment, increasing healthcare burden and costs [3]. HNC management includes surgery, radiotherapy, immunotherapy, chemotherapy, or combined treatments according to disease stage [4]. Radiation toxicity affects quality of life and may lead to treatment discontinuation [3].

Management of HNC include surgery, radiotherapy, immunotherapy, chemotherapy or combination of any strategy according to the stage [4]. The adverse event associated with radiation significantly influences the quality of life of HNC patients and may precipitate the cessation of treatment [5]. Notably, radiotherapy is known to elicit mucositis and dysphagia, alongside xerostomia and dysgeusia, ultimately resulting in malnutrition and subsequent weight loss [6]. Furthermore, malnutrition is prevalent at the time of diagnosis in 30–50% of HNC patients and may exacerbate throughout the treatment regimen [7].

The 8^TH^ edition TNM staging system, human papillomavirus (HPV) status, and patient factors such as smoking, alcohol use, performance status, and age are important prognostic factors for HNC [8, 9]. Recent research in genetics, epigenetics, and OMICs (such as radiomics, metabolomics, and dosiomics) aims to improve risk assessment and treatment response in HNC patients [10–13]. Conversely, there is a dearth of predictive factors indicating severe treatment-related adverse event, particularly radiation-induced adverse event, which are largely confined to dosimetric variables closely associated with treatment planning in radiotherapy [14]. Furthermore, several models estimate the risk of normal tissue complications using NTCP to assess radiation-related side effects [15]. Recent advances in radiomics and imaging help identify patients at risk of radiation adverse event early [16, 17]. Many cancer survivors also face financial and time burdens from diagnosis and treatment, which can reduce quality of life and worsen outcomes [18, 19].

The financial strain faced by patients with HNC is particularly pertinent, considering that this type of malignancy necessitates extensive healthcare interventions and can lead to enduring functional impairments, including those affecting speech, aesthetics, or swallowing [20]. Research involving HNC patients has shown that financial adverse event correlates with decreased overall survival and cancer-specific survival rates [21].

The aim of this particular research endeavor was to thoroughly investigate and critically analyze the various dose rates and their significant influence on the manifestation of toxic effects in head and neck cancer patients, thereby contributing to a deeper understanding of how different levels of dose rate can lead to adverse health outcomes.

## Patients and methods

This study was designed as a prospective pilot investigation at Ayadi Al-Mostakbal Oncology Hospital from December 2023 until May 2025, involving 60 patients divided into three groups. The first group received 600 dose rate FFF 6MV IMRT technique, the second group treated with the 1000 dose rate FFF 6MV IMRT, and the final group underwent 1400 dose rate FFF 6MV IMRT technique. The study’s inclusion criteria were rigorously defined to uphold the validity and reliability of its findings. Participants were required to have pathologically confirmed Primary head and neck squamous cell carcinoma, specifically Stage III-IVB (T3-4 N1-3 M0), and to be adults over 18 years old. Furthermore, the cohort consisted of patients indicated for definitive radiotherapy concurrent with chemotherapy (Cisplation weekly 40mg/m^2^) utilizing intensity-modulated radiation therapy (IMRT), with an assessment of adverse event throughout treatment and a minimum follow-up of four months post-therapy for detection of tumor response by imaging. Exclusion criteria were also carefully established, eliminating patients with metastasis, those over 75 years old, contraindications to radiotherapy, prior malignancies, history of previous chemotherapy or radiotherapy, and incomplete or partial responses to the radiotherapy course.

Following the acquisition of informed consent, patients were prepared for radiotherapy, with demographic data was collected. Each patient was securely immobilized using a neck support and aquaplast mask, followed by CT scans of the head and neck captured via a Simulator CT apparatus, and the images were processed in the eclipse treatment planning system. The treatment plan, which included for each patient, delineation of multiple planning target volumes (PTVs) were determined based on the patient’s diagnosis, including PTV low risk (54 Gy), PTV intermediate risk (60 Gy), and PTV high risk (70GY), each with specific dose ranges and expansion margins. The PTVs, along with critical organs such as the brain stem, parotid glands, oral cavity, and spinal cord, were outlined by the physician, and doses were delivered using a sequential boost technique., was verified by a radiation oncology specialist, then the planning process was conducted by a qualified medical physicist employing the treatment planning system. The total prescribed dose for all plans was 70 Gy (Gy) in 35 fractions. Each treatment plan was meticulously reviewed by the radiation oncology consultant. Upon receiving approval for the plan, treatments were administered via a true beam varian linear accelerator with 6FFF MV photons.

The Sequential Boost Technique is implemented for the treatment regimen, commencing with phase (1) which encompasses a cumulative dose of 54 Gy administered in 27 fractions targeting the PTV categorized as low risk; subsequently, phase (2) entails a total dose of 6 Gy delivered in 3 fractions directed towards the PTV identified as intermediate risk; finally, phase (3) consists of a total dose of 10 Gy allocated in 5 fractions for the PTV classified as high risk.

Throughout the course of IMRT period, acute adverse events; which encompass a range of adverse effects such as weight loss, mucositis, dermatitis, xerostomia, dysphagia, anorexia, and dysgeusia, were systematically monitored weekly by radiation oncology specialist. All adverse events were classified according to the Common Terminology Criteria for Adverse Events (CTCAE) scale [22], thus ensuring comprehensive insights into treatment efficacy and adverse effects.

The inquiries were posed by the radiation oncologist, with all reported discomfort being rigorously evaluated and categorized in accordance with the predefined criteria established in the EORTC (European Organization for Research and Treatment of Cancer) scale. The QLQ-H&N 35, developed by the EORTC; the numerical scale implemented in this study is designed to categorize symptom severity, assigning a score of 1 to indicate a complete absence of symptoms (not at all), a score of 2 to reflect the presence of mild symptoms (a little), a score of 3 to represent moderate symptoms (quite a bit), and a score of 4 to denote the experience of severe symptoms (very much). To ensure the reliability and validity of the data collected, we engaged all participating patients in this investigation by posing the same set of questions three distinct times; the first interval occurred during the period spanning at week two, the second assessment took place at week four, and the final evaluation was conducted at week seven. The comprehensive questionnaire utilized in this study comprises a total of 33 questions designed to capture the nuances of patients’ experiences and symptomatology during their treatment journey. This methodological approach not only facilitates a thorough understanding of the symptom dynamics experienced by patients but also enhances the robustness of the findings by allowing for temporal comparisons. Ultimately, this study aims to contribute significant insights into the management of symptoms in head and neck cancer patients undergoing radiotherapy, thereby informing clinical practice and potential interventions.

Data validation and analysis were performed using SPSS version 22.0. The assessment of all continuous variables for normality was conducted employing the Shapiro-Wilk test. In cases where the variables exhibited a normal distribution, they were reported as Mean ± Standard deviation; otherwise, they were presented as Median (Interquartile range). All categorical variables were reported in terms of either percentages or proportions. The Kruskal-Wallis H test was employed for the comparison of non-normally distributed continuous variables. For categorical variables, comparisons were executed using either the chi-square test or Fisher’s exact test, contingent upon the sample size. A p-value of less than 0.05 was deemed statistically significant.

## Results

### Patient characteristics

In the framework of our extensive investigation, it has been ascertained that the median age of patients in DR 600 and DR 1000 is 60 years, while in DR 1400, it is 61 years. Additionally, within the study cohort, it is significant to highlight that male subjects constitute a considerable majority, comprising 61.7%, equating to 37 patients, in contrast to female subjects, who represent a markedly smaller fraction at 38.3%, corresponding to 23 patients. Concerning the site of primary malignancy, patients were uniformly among allocated three dose rates categories: the larynx, which encompasses 30% of patients across all groups; the parotid gland, accounting for 5%; the Oral cavity, representing 30%; and the Pharynx, which comprise 35%. In terms of TNM staging, the predominant classification is T3N1, constituting 50% in DR 600, 60% in DR 1000, and 50% in DR 1400. The subsequent most prevalent category is T4N1, representing 30% in DR 600, 20% in DR 1000, and 30% in DR 1400. The dose administered across all patients was 70 Gy utilizing the IMRT technique with Cisplation weekly 40 mg/m2 during the period of radiotherapy course. Upon follow-up at four months, no evidence of local or distant recurrence observed for all the patients as shown in (Table 1).

**Table 1:** Demographic criteria of head and neck cancer patients

Demographic criteria Dose rates	Dose rates			*P* value
	DR600 *n* = 20,** (%)**	DR1000 *n* = 20,** (%)**	DR1400 *n* = 20,** (%)**	
Age, Median (range)	60 (48–70)	60 (43–69)	61 (52–69)	0.846
Sex				0.400
- Male n (%) - Female (%)	14 (70%) 6 (30%)	10 (50%) 10 (50%)	13 (65%) 7 (35%)	
Site of carcinoma				1.000
Larynx	6 (30%)	6 (30%)	6 (30%)	
Parotid	1 (5%)	1 (5%)	1 (5%)	
Oral cavity	6 (30%)	6 (30%)	6 (30%)	
Pharynx	7 (35%)	7 (35%)	7 (35%)	
Stage (TNM)				0.681
T3N1M0	10 (50%)	12 (60%)	10 (50%)	
T4N1M0	6 (30%)	4 (20%)	6 (30%)	
T4N3M0	2 (10%)	0 (0.0%)	2 (10%)	
T2N1MO	2 (10%)	2 (10%)	2 (10%)	
T3N2M0	0 (0.0%)	2 (10%)	0 (0.0%)	
Total resection, n (%)	NO	NO	NO	NS
RT dose, Gy,	70GY	70GY	70GY	NS
Technique	IMRT	IMRT	IMRT	NS
Chemotherapy	Cisplation weekly 40 mg/m2	Cisplation weekly 40 mg/m2	Cisplation weekly 40 mg/m2	NS
Follow-up, months	4–6	4–6	4–6	NS
Relapse pattern				1.000
Locoregional-	0 (0.0%)	0 (0.0%)	0 (0.0%)	
Distant-	0 (0.0%)	0 (0.0%)	0 (0.0%)	
No -	20 (100%)	20 (100%)	20 (100%)	

Abbreviation : *NS *Non-significant

The analysis of means ± standard deviation (SD) of three groups revealed that differences in treatment coverage levels among three dose rate categories across three phases for radiotherapy treatment for head and neck cancer, but these variations lacked statistical significance except for the Monitor Unit (MU) and, Treatment Time (min), DR 600 demonstrated better results for MU, while the DR 1400 demonstrated better results for treatment time, as well as no significant differences in organ dose absorption among three dose rate groups across all treatment phases for head and neck cancer patients, while the combination of low dose rate with IMRT markedly decreased the maximum and mean absorbed doses to critical structures. All other dosemetric comparative parameters were illustrated in (Table 2).

**Table 2:** Mean ± SD of dosimetric parameters of target volume coverage and dose received to organs at risk of phase (1), phase (2), and phase (3) in three different dose rates plans in patients with head and neck cancer.

Head and Neck Cancer Patients (*n* = 60)														
Ph1 low dose (54 Gy)	**PTV 54GY**			**OARs**									**Mointer** **Unit** **(MU)**	**Treatment Time (min)**
	**D**_**Min**_	**D**_**Mean**_	**D**_**Max**_	**BS** **D**_**MAX**_	**SC** **D**_**MAX**_	**M** **D**_**MAX**_	**LB** **D**_**mean**_	**RB** **D**_**mean**_	**OC** **D**_**mean**_	**PC** **D**_**mean**_	**LBP** **D**_**MAX**_	**RBP** **D**_**MAX**_		
DR 600 (Gy)	34.30 ± 8.56	53.85 ± 0.57	57.83 ± 1.04	28.64 ± 15.55	35.18 ± 3.29	54.27 ± 9.13	17.78 ± 2.93	19.18 ± 6.68	24.78 ± 12.34	39.28 ± 4.88	54.36 ± 1.83	54.61 ± 1.46	2660 ± 577	4.43 ± 0.96
DR1000 (Gy)	34.71 ± 8.50	53.85 ± 0.53	57.96 ± 1.158	29.10 ± 15.73	35.46 ± 3.26	54.56 ± 9.14	18.30 ± 2.94	19.58 ± 6.67	25.14 ± 12.37	39.51 ± 4.88	54.39 ± 1.90	54.64 ± 1.58	3110 ± 672	3.11 ± 0.67
DR1400 (Gy)	35.09 ± 8.16	53.92 ± 0.56	58.36 ± 1.21	29.52 ± 15.82	36.06 ± 3.22	54.70 ± 9.13	18.76 ± 3.13	20.03 ± 6.66	25.46 ± 12.31	39.83 ± 4.85	54.42 ± 1.96	54.72 ± 1.55	3538 ± 744	2.53 ± 0.53
*P *value	0.998	0.995	0.256	0.995	0.831	0.997	0.641	0.992	0.996	0.997	0.993	0.998	0.003	0.008

Ph2 intermediate dose (6 Gy)	**PTV 60GY**			**OARs**									**Mointer** **Unit** **(MU)**	**Treatment Time (min)**
	**D**_**Min**_	**D**_**Mean**_	**D**_**Max**_	**BS** **D**_**MAX**_	**SC** **D**_**MAX**_	**M** _**MAX**_	**LB** _**mean**_	**RB** **D**_**mean**_	**OC** **D**_**mean**_	**PC** _**mean**_	**LBP** **D**_**MAX**_	**RBP** **D**_**MAX**_		
DR 600 (Gy)	4.27 ± 1.57	5.96 ± 0.05	6.32 ± 0.18	1.98 ± 1.85	3.23 ± 1.39	5.08 ± 1.95	1.85 ± 1.01	1.66 ± 1.32	2.22 ± 1.84	3.52 ± 1.28	4.91 ± 2.07	4.62 ± 2.15	1537 ± 711	2.56 ± 1.19
DR1000 (Gy)	4.29 ± 1.53	5.96 ± 0.05	6.33 ± 0.19	2.00 ± 1.85	3.25 ± 1.37	5.09 ± 1.94	1.87 ± 1.01	1.68 ± 1.33	2.24 ± 1.84	3.53 ± 1.29	4.93 ± 2.06	4.64 ± 2.13	1804 ± 807	1.80 ± 0.81
DR1400 (Gy)	4.32 ± 1.47	5.96 ± 0.06	6.36 ± 0.22	2.02 ± 1.88	3.29 ± 1.37	5.10 ± 1.96	1.89 ± 1.01	1.69 ± 1.33	2.26 ± 1.83	3.55 ± 1.30	4.95 ± 2.08	4.65 ± 2.18	2067 ± 919	1.48 ± 0.66
*P *value	0.994	1.000	0.929	0.992	0.981	0.994	0.997	0.998	0.994	0.995	0.998	0.991	0.006	0.001

Ph3 high dose (10 Gy)	**PTV 70GY**			**OARs**									**Mointer** **Unit** **(MU)**	**Treatment Time (min)**
	**D**_**Min**_	**D**_**Mean**_	**D**_**Max**_	**BS** **D**_**MAX**_	**SC** **D**_**MAX**_	**M** **D**_**MAX**_	**LB** **D**_**mean**_	**RB** **D**_**mean**_	**OC** **D**_**mean**_	**PC** **D**_**mean**_	**LBP** **D**_**MAX**_	**RBP** **D**_**MAX**_		
DR 600 (Gy)	7.43 ± 2.21	9.98 ± 0.21	10.60 ± 0.28	1.52 ± 2.16	4.92 ± 1.65	5.94 ± 4.35	1.29 ± 1.51	1.44 ± 2.01	2.63 ± 3.32	4.98 ± 1.88	6.32 ± 3.05	6.16 ± 3.04	1255 ± 382	2.09 ± 0.64
DR1000 (Gy)	7.44 ± 2.19	9.99 ± 0.21	10.61 ± 0.28	1.54 ± 2.20	4.94 ± 1.61	5.94 ± 4.37	1.31 ± 1.51	1.45 ± 2.03	2.64 ± 3.32	4.99 ± 1.87	6.34 ± 3.04	6.20 ± 3.05	1453 ± 442	1.45 ± 0.44
DR1400 (Gy)	7.46 ± 2.18	10.02 ± 0.21	10.64 ± 0.29	1.55 ± 2.17	5.01 ± 1.65	6.03 ± 4.41	1.34 ± 1.53	1.48 ± 2.04	2.67 ± 3.35	5.00 ± 1.86	6.38 ± 3.04	6.21 ± 2.99	1652 ± 488	1.18 ± 0.35
*P *value	0.997	0.994	0.996	0.991	0.986	0.992	0.993	0.998	0.994	0.995	0.997	0.996	0.005	0.007

Abbreviations: *BS* Brain stem , *SC *Spinal cord , *M *Mandibule , *LP *LT Parotied , *RP *RT Parotied, *OC *Oral cavity, *PC* Pharyngal constructor , LT Brachial plexus (LBP), and RT Brachial plexus (RBP)

The acute adverse events observed in head and neck cancer patients receiving radiotherapy will be detailed outlined in (Tables 3 and 4).

**Table 3:** Acute adverse events of head and neck cancer patients in period from baseline to week four

weeks		Dose rates	Mucositis			Xerostomia		Dermatitis		dysphagia			Anorexia			dysgeusia	W-Loss
week 1		DR600 (*n* = 20)	G0 (16)	GI (4)		G0		GO		G0 (14)	GI (6)		G0			G0	0 (0–1)
		DR 1000 (*n* = 20)	G0 (10)	GI (10)		G0		GO		G0 (14)	GI (6)		G0			G0	0 (0–2)
		DR 1400 (*n* = 20)	G0 (14)	GI (6)		G0		G0		G0 (16)	GI (4)		G0			G0	0.5 (0–2)
*P *value Of Week (1)			0.122			1.000		1.000		0.711			1.000			1.000	0.474
week 2		DR600 (*n* = 20)	G0 (12)	GI (8)		G0		G0		G0 (10)	GI (10)		G0			G0	1(0–4)
		DR 1000 (*n* = 20)	G0 (8)	GI (12)		G0		G0		G0 (10)	GI (10)		G0			G0	1(1–5)
		DR 1400 (*n* = 20)	G0 (12)	GI (8)		G0		G0		G0 (10)	GI (10)		G0			G0	1(1–5)
*P *value Of Week (2)			0.343			1.000		1.000		1.000			1.000			1.000	0.449
week 3		DR600 (*n* = 20)	GI (14)	GII (6)		G0		G0 (15)	GI (5)	GI (17)	GII (3)		G0 (20)		GI (0)	G0	3 (1–4)
		DR 1000 (*n* = 20)	GI (12)	GII (8)		G0		G0 (14)	GI (6)	GI (17)	GII (3)		G0 (17)		GI (3)	G0	3 (1–7)
		DR 1400 (*n* = 20)	GI (14)	GII (6)		G0		G0 (14)	GI (6)	GI (18)	GII (2)		G0 (18)		GI (2)	G0	5 (1–8)
*P *value Of Week (3)			0.741			1.000		0.921		0.866			0.217			1.000	0.028
week 4	DR600 (*n* = 20)		GI (8)		GII (12)	G0 (20)	GI (0)	G0 (15)	GI (5)	GI (13)		GII (7)	G0 (20)	GI (0)		G0	4 (3–7)
	DR 1000 (*n* = 20)		GI (8)		GII (12)	G0 (18)	GI (2)	G0 (11)	GI (9)	GI (16)		GII (4)	G0 (16)	GI (4)		G0	4 (2–7)
	DR 1400 (*n* = 20)		GI (4)		GII (16)	G0 (18)	GI (2)	G0 (10)	GI (10)	GI (13)		GII (7)	G0 (18)	GI (2)		G0	5.5 (2–8)
*P *value Of Week (4)			0.301			0.343		0.233		0.495			0.108			1.000	0.255

The analysis in (Table 3) revealed no statistically significant variation in parameters such as mucositis, dermatitis, xerostomia, dysphagia, anorexia, dysgeusia, and weight loss during the first 20 fractions of radiotherapy for head and neck cancer patients; however, a significant weight loss difference (*p* = 0.028) was observed at third week for patients receiving the 600dose rate. So that dose rate alterations do not affect radiation-induced adverse event at this treatment stage.

**Table 4:** Acute adverse events of head and neck cancer patients in period from week five to week seven

weeks	Dose rates	Mucositis		Xerostomia			Dermatitis			dysphagia			Anorexia				dysgeusia	W-Loss
week 5	DR600 (n=20)	GI(6)	GII (14)	G0(20)	GI(0)		G0(10)	GI(10)		GI(12)	GII (8)		G0 (18)		GI (2)		G0	5 (3-8)
	DR 1000 (n=20)	GI(5)	GII (15)	G0(18)	GI(2)		G0(10)	GI(10)		GI(8)	GII (12)		G0(15)		GI (5)		G0	6 (3-9)
	DR 1400 (n=20)	GI(4)	GII (16)	G0(17)	GI(3)		G0(8)	GI(12)		GI(4)	GII (16)		G0(11)		GI (9)		G0	7 (4-10)
*P *value Of Week (5)		0.766		0.217			0.002			0.036			0.043				1.000	0.002
week 6	DR600 (n=20)	GII (16)	GIII(4)	G0(15)	GI(5)		G0(11)	GI(8)	GII(1)	GI(10)	GII (10)		G0 (16)		GI (4)		G0	6 (4-11)
	DR 1000 (n=20)	GII (14)	GIII(6)	G0(10)	GI(10)		G0(5)	GI(13)	GII(2) (13)	GI(4)	GII (16)		G0(13)		GI (7)		G0	7 (5-10)
	DR 1400 (n=20)	GII (12)	GIII(8)	G0(7)	GI(13)		G0(5)	GI(8)	GII (7)	GI(2)	GII (18)		G0(8)		GI (12)		G0	8 (7-12)
*P *value Of Week (6)		0.386		0.038			0.023			0.012			0.032				1.000 11 00861.000 1.000 1.000	0.001
week 7	DR600 (n=20)	GII (14)	GIII(6)	G0(11)	GII (8)	GIII(1)	G0(14)	GI(5)	GII(1)	GI(7)	GII(7)	GIII(6)	G0(14)	GI(5)	GII(1)	GIII(0)	G0	6.5 (4-14)
	DR 1000 (n=20)	GII (10)	GIII(10)	G0(4)	GII (14)	GIII(2)(2) (13)	G0(4)	GI(14)	GII(2) (13)	GI(2)	GII(14) (13)	GIII(4)(2) (13)	G0(6)	GI(10)	GII(2) (13)	GIII(2)(2) (13)	G0	7.5 (6-12)
	DR 1400 (n=20)	GII (9)	GIII(11)	G0(0)	GII(13)	GIII(7)	G0(5)	GI(6)	GII (9)	GI(0)	GII (13)	GIII(7)	G0(8)	GI(0)	GII (6)	GIII(6)	G0	9 (7-14)
*P *value Of Week (7)		0.243		0.002			0.000			0.017			0.000				1.000	0.019

Further examination of acute adverse event at week five demonstrated no statistically significant variations in mucositis, xerostomia, and dysgeusia among the dose rates of DR600, DR1000, and DR1400 (*P* = 0.766, *P* = 0.217, *P* = 1.000); however, an elevation in radiation dose rates was associated with notable increments in dermatitis, dysphagia, anorexia, and weight loss, substantiated by p-values of (*P* = 0.002, *P* = 0.036, *P* = 0.043, *P* = 0.002). The findings of the investigation suggested the absence of significant differences in mucositis and dysgeusia among the three groups at week six (*P* = 0.386 and *P* = 1.000), yet a heightened radiation dose was significantly correlated with substantial increases in xerostomia, dermatitis, dysphagia, anorexia, and weight loss (*P* = 0.038, *P* = 0.023, *P* = 0.012, *P* = 0.032, and *P* = 0.001). The continued investigation at week seven indicated no significant differences in mucositis and dysgeusia among the three groups (*P* = 0.243 and *P* = 1.000), yet an augmented radiation dose rate was significantly correlated with increased occurrences of xerostomia, dermatitis, dysphagia, anorexia, and weight loss, as evidenced by p-values of (*P* = 0.002, *P* = 0.000, *P* = 0.017, *P* = 0.000, and *P* = 0.019), as represented in (Table 4).

The study revealed significant adverse event changes in the fifth to seventh weeks. The findings emphasize the necessity for ongoing evaluation and management of acute side effects as treatment progresses as shown in (Table 4).

**Fig. 1:**
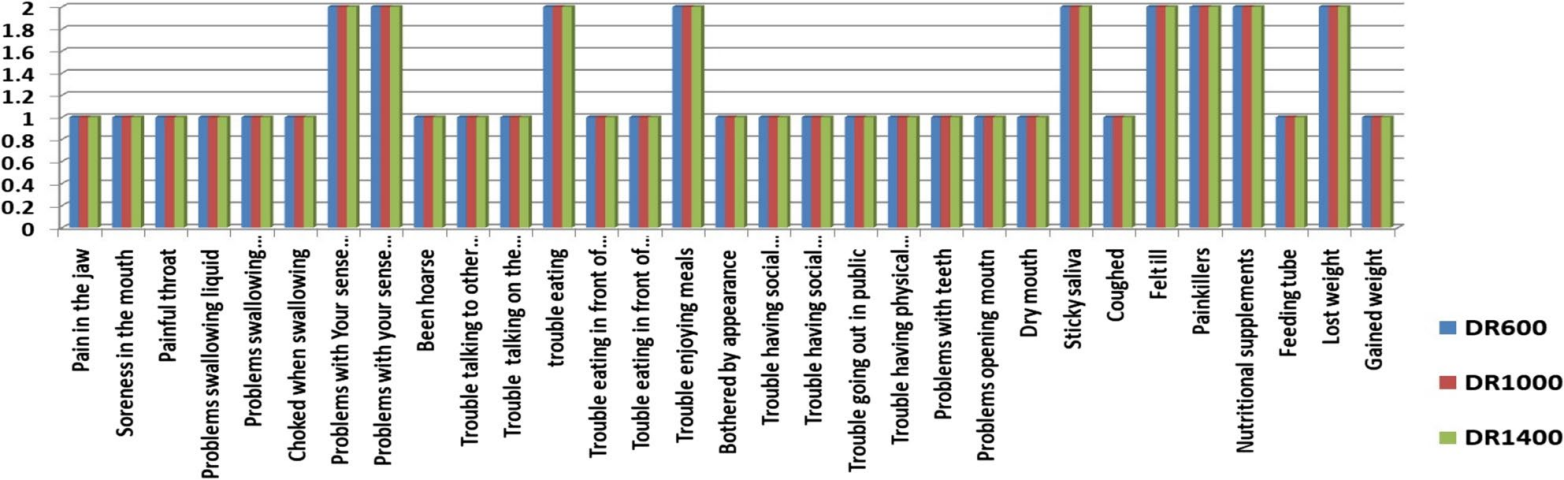
The symptoms or problems in QLQ-H&N 35 of head and neck cancer patients at week two. Patients revealed a numerical scale for symptom severity, from 1 (not at all) to 2 (a little)

**Fig. 2:**
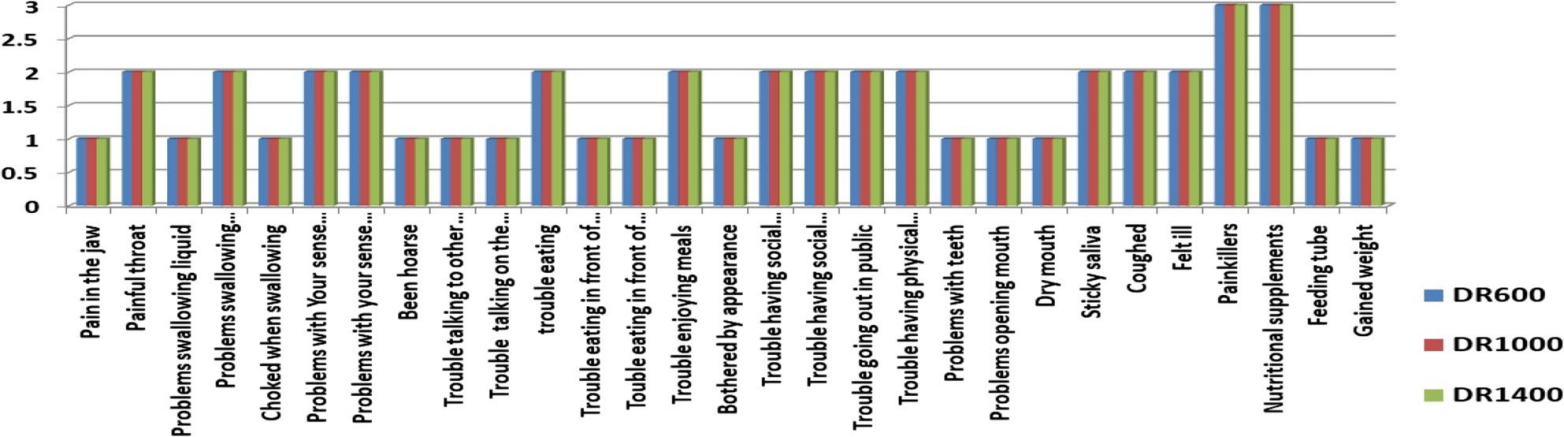
The symptoms or problems in QLQ-H&N 35 of head and neck cancer patients at week four. Patients revealed a numerical scale for symptom severity, from 1 (not at all) to 3 (quite a bit)

**Fig. 3:**
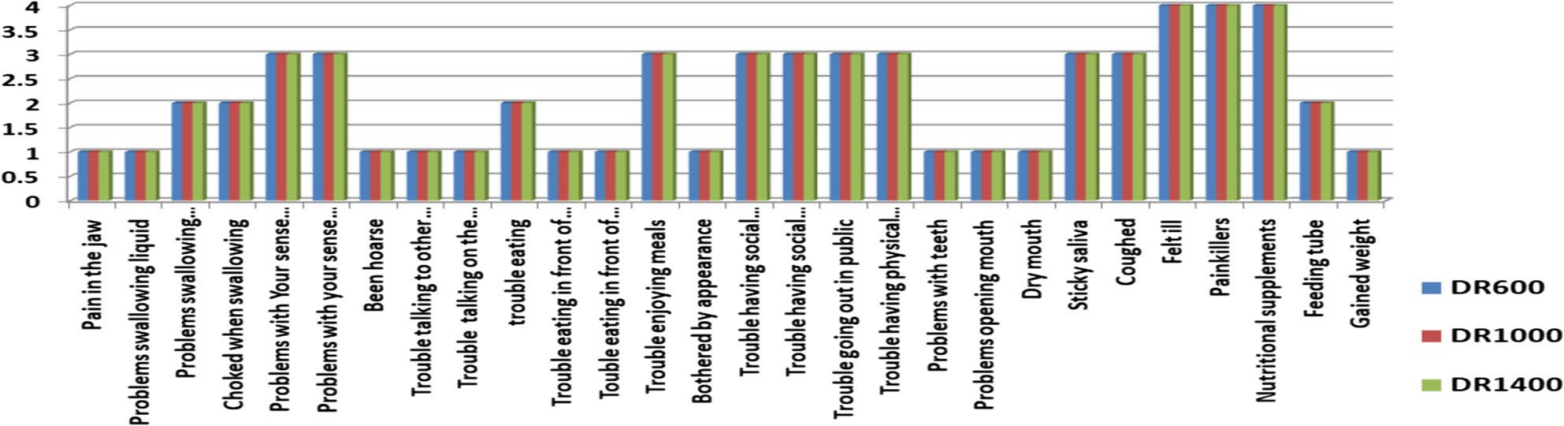
The symptoms or problems in QLQ-H&N 35 of head and neck cancer patients at week seven. Patients revealed a numerical scale for symptom severity, from 1 (not at all) to 4 (very much)

In the detailed examination and comprehensive analysis presented in (Table 5) alongside the illustrative representations found in figures ranging from (Figs. 1, 2, and 3) it is imperative to underscore that at week two only two out of thirty-three symptoms displayed variability across three dose rate groups, with all observed differences being statistically insignificant, while patients reported symptom severity scores ranging from 1 (not at all) to 2 (a little). Furthermore, at week four, only four out of thirty-three symptoms varied among dose rate groups, with a statistically significant p-value of 0.036 for weight loss, while patient-reported severity ranged from 1 (not at all) to 3 (quite a bit); similarly, at week seven, six symptoms showed variability, yet only weight loss maintained a significant p-value of 0.003, with severity ratings extending from 1 (not at all) to 4 (very much).

**Table 5:** The symptoms or problems in QLQ-H&N 35 of head and neck cancer

SymptomsAt week two	DR600n=20, (%)			DR1000n=20, (%)				DR1400n=20, (%)			*P *value
	1	2		1	2			1	2		
Pain in the mouth	18 (90%)	2 (10%)		16 (80%)	4 (20%)			12 (60%)	8 (40%)		0.074
	1	2		1	2			1	2		
Problems swallowing solid food	16 (80%)	4 (20%)		14 (70%)	6 (30%)			12 (60%)	8 (40%)		0.386

SymptomsAt week four	DR600n=20, (%)			DR1000n=20, (%)				DR1400n=20, (%)			*P *value
	2	3		2	3			2	3		
Pain in the mouth	18 (90%)	2 (10%)		16 (80%)	4 (20%)			14 (70%)	6 (30%)		0.287
	1	2		1	2			1	2		
Soreness in the mouth	20 (100%)	0 (0.0%)		17 (85%)	3 (15%)			16 (8O%)	4 (20%)		0.122
	2	3		2	3			2	3		
Problems swallowing solid food	10 (50%)	10 (50%)		9 (45%)	11 (55%)			8 (40%)	12 (60%)		0.817
	1	2	3	1	2	3		1	2	3	
Lost weight	2 (10%)	12 (60%)	6 (30%)	0 (0.0%)	14 (70%)	6 (30%)		0 (0.0%)	7 (35%)	13 (65%)	0.036

SymptomsAt week seven	DR600 n=20, (%)			DR1000n=20, (%)				DR1400n=20, (%)			*P *value
	2	3	4	2	3	4	2		3	4	
Pain in the mouth	10 (50%)	10 (50%)	0 (0.0%)	9 (45%)	9 (45%)	2 (10%)	8 (40%)		9 (45%)	3 (15%)	0.542
	1	2	3	1	2	3	1		2	3	
Soreness in the mouth	20 (100%)	0 (0.0%)	0 (0.0%)	14 (70%)	4 (20%)	2(10%)	13 (65%)		5(25%)	2(10%)	0.075
	2	3		2	3			2	3		
Painful throat	16 (80%)	4 (20%)		14 (70%)	6 (30%)			10 (50%)	10 (50%)		0.122
	3	4		3	4			3	4		
Problems swallowing solid food	12 (60%)	8 (40%)		12 (60%)	8 (40%)			12 (60%)	8 (40%)		1.000
	1	2		1	2			1	2		
Dry mouth	8 (40%)	12 (60%)		2 (10%)	18 (90%)			4 (20%)	16 (80%)		0.074
	2	3	4	2	3	4		2	3	4	
Lost weight	10 (50%)	6 (30%)	4 (20%)	2 (10%)	12 (60%)	6 (30%)		1 (5%)	9 (45%)	10 (50%)	0.003

## Discussion

The management of HNC presents unique challenges due to aggressive treatment regimens that adversely affect patients’ QoL, necessitating a comprehensive understanding by healthcare professionals to enhance supportive care and patient outcomes [23]. To our knowledge, this is the first study to analysis of the effects of dose rate on external beam radiation therapy employing the free flattening filter (FFF) technique, which is already integrated into clinical practice.

While previous studies have explored FLASH irradiation in ultra-high dose rate radiotherapy [24], the clinical application of FFF beams necessitates an evaluation of their effects on antitumor immune responses relative to standard low dose rate radiotherapy, particularly in light of the growing number of clinical trials involving immune-checkpoint blockade [25], with the dose-rate effect demonstrating that high dose rates enhance biological efficacy and cell death, though its actual influence is complex and varies by tissue type rather than conforming to fixed classifications [26].

Prior to commencing treatment, oncologists must engage in thorough discussions with patients regarding critical guidelines, including the importance of hydration and the avoidance of hot and spicy foods for comfort. Furthermore, the implementation of supportive mouthwash and antifungal medication, alongside nutritional support during radiotherapy treatment course, exemplifies the comprehensive approach required to optimize patient well-being and therapeutic effectiveness during oncological care.

The study conducted by van der Laan HP et al. [27] was to elucidate the differential effects of physician-assessed toxicities and patient-reported symptoms in HNC on QOL, alongside evaluating these factors for the enhancement and selection of treatment strategies. This prospective cohort study involved 1,083 HNC patients undergoing definitive radiotherapy, with assessments conducted at various intervals post-treatment. The findings indicated that radiation-induced adverse events precipitated a significant decrement in QOL, with specific symptoms such as xerostomia and weight loss exhibiting minor effects, whereas issues like speech impairment and fatigue demonstrated a more pronounced impact on patient well-being.

Previous studies [28] have revealed an ambiguity surrounding the determinants influencing the manifestation of radiation-induced adverse event, and they have further elucidated that such toxicities have a direct impact on the QOL of patients. Consequently, the objective of our research was to elucidate the influence of radiation dose rate on the adverse events elicited by radiation exposure, for a more profound and comprehensive understanding of radiotherapy and its implications concerning adverse events.

In our study, the cases were distributed across three groups to eliminate confounding variables regarding tumor origin, revealing no statistically significant differences in demographic and clinical parameters such as age (*p* = 0.846), gender (*p* = 0.400), and TNM stage (*p* = 0.681), thereby affirming the groups’ homogeneity for a more accurate assessment of treatment-related adverse event outcomes, as shown in (Table 1).

The results of the present study indicated that, there are no statistically significant differences in target volumes coverage and critical organs radiation absorption across three dose rate categories in head and neck cancer radiotherapy, although an increase in radiation dose rate correlates with a higher maximum and mean absorbed dose in various organs at risk without any statistically significant differences among the three groups in the three phases of treatment, as detailed in (Table 2).

Our study presents a comparative examination of acute radiation-induced adverse events, indicating no statistically significant differences in mucositis and dysgeusia, as well as other parameters, among the treatment groups during the initial four-week period; nevertheless, from week five to week seven, elevated dose rates were correlated with an increase in Xerostomia, dermatitis, dysphagia, anorexia, and weight loss, as substantiated by the data illustrated in (Tables 3 and 4).

The assessment of acute radiation adverse event during a seven-week radiotherapy regimen indicates that the radiation dose rate significantly impacts treatment outcomes from the fifth to the seventh week, suggesting that for head and neck cancer patients, a low dose rate is preferable, although a high dose rate may be utilized in the initial 20 fractions if patient volume necessitates expedited treatment, followed by a shift to a lower dose rate to reduce radiation adverse event, However additional trials are requisite to substantiate the findings, particularly concerning the evaluation of late toxicity.

Upon analyzing the QLQ-H&N 35 for head and neck cancer patients, it is imperative to underscore that notwithstanding the discernible divergences in symptomatology, a limited number of symptoms exhibit variation across the three distinct groups characterized by different dose rates, but these differences did not attain statistical significance. Consequently, the dose rate seems to exert no significant influence on the QoL during the period of radiotherapy course treatment.

Among the constraints, one must note the limited patient cohort and the brief follow-up period. Additionally, we did not examine the adverse event profile three months post-treatment, which could have facilitated a more comprehensive understanding of the trends in adverse events, whether amelioration or persistence. The evaluation of adverse events at seven weeks did not incorporate the assessment of vocal quality following the intervention. We are committed to addressing these limitations by integrating these aspects into our forthcoming research endeavors.

In conclusion, more research is needed to confirm the findings of this study. Future studies should include a diverse population of individuals affected by HNC to validate these conclusions. Additionally, replicating this study with a larger sample size is important to ensure the reliability of the results.

## Conclusion

This study demonstrates that the dose rate seemingly plays a significant role in determining the overall health outcomes of patients, although it does not appear to have a substantial effect on the quality of life (QoL) experienced by those individuals undergoing radiotherapy treatment of head and neck cancer patients.

## Data Availability

Data supporting the findings of this study are available from the corresponding author upon reasonable request.

## Abbreviations

QOL: Quality of Life
HNC: Head and neck cancer
HPV: Human papillomavirus
NTCP: Normal Tissue Complication Probability
FFF: Flattening Filter–Free
IMRT: Intensity–Modulated Radiation Therapy
PTVs: Planning target volumes Gy–Gray
CTCAE: Common Terminology Criteria for Adverse Events
EORTC: European Organization for Research and Treatment of Cancer
MUs: Monitor Units
SD: standard deviation

## References

[1] Nigro CL, Denaro N, Merlotti A, Merlano M. Head and neck cancer: improving outcomes with a multidisciplinary approach.Cancer Manag. Res. 2017;9:363–71. 10.2147/CMAR.S115761 PMC5571817 28860859

[2] Cramer JD, Burtness B, Le QT, Ferris RL. The changing therapeutic landscape of head and neck cancer. Nat Rev Clin Oncol. 2019;16:669–83. 31189965 10.1038/s41571-019-0227-z

[3] Pollaers K, Massingham I, Friedland PL, Farah CS. The economic burden of oral squamous cell carcinoma in Australia.J. Oral Pathol. Med. 2019;48:588–94. 10.1111/jop.12907 31177557

[4] Pignon JP, le Maître A, Maillard E, Bourhis J, MACH-NC Collaborative Group. Meta-analysis of chemotherapy in head andneck cancer (MACH-NC): an update on 93 randomised trials and 17,346 patients. Radiother Oncol. 2009;92:4–14. 19446902 10.1016/j.radonc.2009.04.014

[5] Van den Bosch L, van der Laan HP, van der Schaaf A, Oosting SF, Halmos GB, et al. Patient-reported toxicity and quality-of-life profiles in patients with head and neck cancer treated with definitive radiation therapy or chemo radiation. Int J Radiat Oncol Biol Phys. 2021;111(2):456–67. 34048816 10.1016/j.ijrobp.2021.05.114

[6] Mason H, DeRubeis MB, Burke N, Shannon M, Karsies D, Wolf G, Eisbruch A, Worden F. Symptom management duringand after treatment with concurrent chemoradiotherapy for oropharyngeal cancer: a review of the literature and areas for futureresearch. World J Clin Oncol. 2016;7:220–6. 27081644 10.5306/wjco.v7.i2.220 PMC4826967

[7] Magnano M, Mola P, Machetta G, Maffeis P, Forestiero I, Cavagna R, Artino E, Boffano P. The nutritional assessment ofhead and neck cancer patients. Eur Arch Oto-Rhino-Laryngol. 2015;272:3793–9. 10.1007/s00405-014-3462-z 25534287

[8] Huang SH, O’Sullivan B. Overview of the 8th edition TNM classification for head and neck cancer. Curr Treat Options Oncol. 2017;18:40. 28555375 10.1007/s11864-017-0484-y

[9] Argiris A, Li Y, Forastiere A. Prognostic factors and long-term survivorship in patients with recurrent or metastatic carcinomaof the head and neck. Cancer. 2004;15:2222–9. 10.1002/cncr.20640 15452834

[10] Furlan C, Polesel J, Barzan L, Franchin G, Sulfaro S, Romeo S, Colizzi F, Rizzo A, Baggio V, Giacomarra V, et al. Prognostic significance of LINE-1 hypomethylation in oropharyngeal squamous cell carcinoma. Clin Epigenetics. 2017;9:58. 28572862 10.1186/s13148-017-0357-z PMC5450111

[11] Avanzo M, Stancanello J, El Naqa I. Beyond imaging: the promise of radiomics. Phys Med. 2017;38:122–39. 28595812 10.1016/j.ejmp.2017.05.071

[12] Gabry´s HS, Buettner F, Sterzing F, Hauswald H, Bangert M. Design and selection of machine learning methods usingradiomics and dosiomics for normal tissue complication probability modeling of xerostomia. Front Oncol. 2018;8:35. 29556480 10.3389/fonc.2018.00035 PMC5844945

[13] Tan FH, Bai Y, Saintigny P, Darido C. mTORsignalling in head and neck cancer: Heads up. Cells. 2019;8:333. 30970654 10.3390/cells8040333 PMC6523933

[14] Bentzen SM, Constine LS, Deasy JO, Eisbruch A, Jackson A, Mark LB, Ten Haken RK, Yorke ED. Quantitative analysesof normal tissue effects in the clinic (QUANTEC): an introduction to the scientific issues. Int J Radiat Oncol Biol Phys. 2010;76:S3–9. 20171515 10.1016/j.ijrobp.2009.09.040 PMC3431964

[15] Van den Bosch L, van der Schaaf A, van der Laan HP, Hoebers FJP, Wijers OB, van den Hoek JGM, Moons KGM, Reitsma JB, Steenbakkers RJHM, Schuit E. Comprehensive toxicity risk profiling in radiation therapy for head and neckcancer: a new concept for individually optimised treatment. Radiother Oncol. 2021;157:147–54. 33545258 10.1016/j.radonc.2021.01.024

[16] Desideri I, Loi M, Francolini G, Becherini C, Livi L, Bonomo P. Application of radiomics for the prediction of radiationinducedtoxicity in the IMRT era: Current state-of-the-art. Front Oncol. 2020;10:1708. 33117669 10.3389/fonc.2020.01708 PMC7574641

[17] Mierzwa ML, Gharzai LA, Li P, Wilkie JR, Hawkins PJ, Aryal MP, Lee C, Rosen B, Lyden T, Blakely A et al. Early MRI blood volume changes in constrictor muscles correlate with postradiation dysphagia. Int. J. Radiat. Oncol. Biol. Phys.2021, in press. 10.1016/j.ijrobp.2020.12.018 PMC12273593 33346093

[18] Zafar SY. Financial toxicity of cancer care: it’s time to intervene. Gynecol Oncol. 2016;108:djv370. 10.1093/jnci/djv370 26657334

[19] Gordon LG, Merollini K, Lowe A, Chan R. A systematic review of financial toxicity among cancer survivors: we can’t paythe co-pay. Patient. 2017;10:295–309. 27798816 10.1007/s40271-016-0204-x

[20] Massa ST, Osazuwa-Peters N, Boakye EA, Walker RJ, Ward GM. Comparison of the financial burden of survivors of headand neck cancer with other cancer survivors. JAMA Otolaryngol Head Neck Surg. 2019;145:239–49. 30789634 10.1001/jamaoto.2018.3982 PMC6439752

[21] Ma SJ, Iovoli AJ, Attwood K, Wooten KE, Arshad H, Gupta V, McSpadden RP, Kuriakose MA, Markiewicz MR, et al. Association of significant financial burden with survival for head and neck cancer patients treated with radiationtherapy. Oral Oncol. 2021;115:105196. 33578203 10.1016/j.oraloncology.2021.105196 PMC10353569

[22] Colevas AD, Lira RR, Colevas EA, Lavori PW, Chan C, Shultz DB, Chang KW. Hearing evaluation of patients with head and neck cancer: comparison of common terminology criteria for adverse events, brock and chang adverse event criteria in patients receiving cisplatin. Head Neck. 2015;37(8):1102–7. 24737682 10.1002/hed.23714 PMC4780572

[23] Crawford KL, Stramiello JA, Orosco RK, Greene JJ. Advances in facial nerve management in the head and neck cancer patient. CurrOpinOtolaryngol Head Neck Surg. 2020;28(4):235–40. 10.1097/MOO.0000000000000641. 10.1097/MOO.0000000000000641 32628417

[24] Jin JY, Gu A, Wang W, Oleinick NL, Machtay M, Spring Kong FM. Ultra-high dose rate effect on circulating immune cells: a potential mechanism for FLASH effect? RadiotherOncol. 2020; 149:55–62. 10.1016/j.radonc.2020.04.054 10.1016/j.radonc.2020.04.054 PMC7442672 32387486

[25] Misaki S, Murata S, Shimoji M, et al. Enhancement of antitumor immune response by radiation therapy combined with dual immune checkpoint inhibitor in a metastatic model of HER2-positive murine tumor. Jpn J Radiol. 2022;40(12):1307–15. 10.1007/s11604-022-01303-z. 35763240 10.1007/s11604-022-01303-z PMC9719888

[26] Favaudon V, Caplier L, Monceau V, et al. Ultrahigh dose-rate FLASH irradiation increases the differential response between normal and tumor tissue in mice. Sci Transl Med. 2014;6:245ra93. 10.1126/scitranslmed.3008973. 25031268 10.1126/scitranslmed.3008973

[27] van der Laan HP, Van den Bosch L, Schuit E, Steenbakkers RJHM, van der Schaaf A, Langendijk JA. Impact of radiation-induced toxicities on quality of life of patients treated for head and neck cancer. Radiother Oncol. 2021;160:47–53. 10.1016/j.radonc.2021.04.011. 33892023 10.1016/j.radonc.2021.04.011

[28] Carbonara R, Bonomo P, Di Rito A, Didonna V, Gregucci F, Ciliberti MP, et al. Investigation of radiation-induced toxicity in head and neck cancer patients through radiomics and machine learning: a systematic review. J Oncol. 2021;2021:5566508. 10.1155/2021/5566508. 34211551 10.1155/2021/5566508 PMC8211491

